# The Polybrominated Diphenyl Ether Bromoxib Disrupts Nuclear Import and Export by Affecting Nucleoporins of the Nuclear Pore Complex

**DOI:** 10.3390/md23030108

**Published:** 2025-02-28

**Authors:** Karina S. Krings, Anastasia Ritchie, Laura Schmitt, Judith Hatzfeld, Gudrun Totzke, Thomas Lenz, María José Mendiburo, Björn Stork, Nicole Teusch, Peter Proksch, Kai Stühler, Lisa Müller, Sebastian Wesselborg

**Affiliations:** 1 Institute for Molecular Medicine I, Medical Faculty and University Hospital Duesseldorf, Heinrich Heine University Duesseldorf, Universitaetsstraße 1, 40225 Duesseldorf, Germany; karina.krings@uni-duesseldorf.de (K.S.K.); laura-schmitt9@web.de (L.S.);; 2 Institute of Virology, Medical Faculty and University Hospital Duesseldorf, Heinrich Heine University Duesseldorf, Universitaetsstraße 1, 40225 Duesseldorf, Germany; anastasia.ritchie@hhu.de (A.R.);; 3 Molecular Proteomics Laboratory, Biological-Medical-Research Center (BMFZ), Medical Faculty and University Hospital Duesseldorf, Heinrich Heine University Duesseldorf, Universitaetsstraße 1, 40225 Duesseldorf, Germany; 4 Institute of Pharmaceutical Biology and Biotechnology, Faculty of Mathematics and Natural Sciences, Heinrich Heine University Duesseldorf, Universitaetsstraße 1, 40225 Duesseldorf, Germany; nicole.teusch@uni-duesseldorf.de (N.T.);; 5 Center for Integrated Oncology Aachen-Bonn-Cologne-Duesseldorf (CIO ABCD), 40225 Duesseldorf, Germany

**Keywords:** marine sponge-derived natural products, polybrominated diphenyl ethers (PBDEs), nucleoporins (NUPs), nuclear pore complex (NPC), nuclear import and export

## Abstract

Polybrominated diphenyl ethers (PBDEs) are natural products with potent antimicrobial and antineoplastic activity. We have previously shown that the polybrominated diphenyl ether bromoxib (4,5,6-tribromo-2-(2′,4′-dibromophenoxy) phenol), isolated from the marine sponge *Dysidea* species, exhibits a strong cytotoxic potential in leukemia and lymphoma cells by targeting mitochondrial metabolism. Here, using a mass spectrometric thermal proteome profiling (TPP) approach, we observed that bromoxib induces a rapid reduction in the levels of 19 nucleoporins (NUPs) that are part of the nuclear pore complex (NPC). This apparently affected the functionality of the NPC, as evidenced by the bromoxib-mediated inhibition of the nuclear translocation and subsequent gene reporter activity of transcription factors such as nuclear factor of activated T cells (NFAT) and nuclear factor κB (NF-κB). In addition, bromoxib inhibited the nuclear export of the mRNA of the human immunodeficiency virus transactivator of transcription (HIV-Tat) and the subsequent import of the HIV-Tat protein into the nucleus as determined by the decrease in Tat-dependent gene reporter luciferase activity. Inhibition of nuclear mRNA-export also affected expression of the short-lived anti-apoptotic Bcl-2 protein Mcl-1, which has been shown to induce apoptosis. Thus, its ability to target both mitochondrial metabolism and the NPC renders bromoxib a promising anticancer agent.

## 1. Introduction

**Figure 1:**
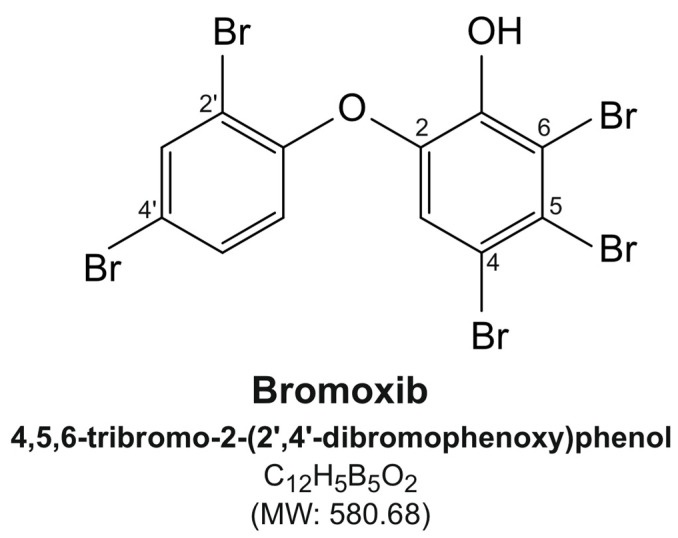
Structure of bromoxib (4,5,6-tribromo-2-(2′,4′-dibromophenoxy)phenol).

Natural marine polybrominated diphenyl ethers (PBDEs) originate from the symbiosis between marine sponges and cyanobacteria or bacteria [1]. They were first isolated from marine sponges of *Dysidea* species in 1981 [2] and comprise diphenyl ether scaffolds and hydroxy (-OH) or methoxy (-MeO), ether, and bromine (-Br) functional groups [3,4]. The polybrominated diphenyl ether bromoxib (4,5,6-tribromo-2-(2′,4′-dibromophenoxy) phenol; Figure 1; for ^1^H-NMR spectrum, see Supplemental Figure S1) was isolated from a marine sponge belonging to the *Dysidea* species [2,5].

We initially identified bromoxib (formerly termed P01F08) in a cytotoxicity screen of 300 natural compounds [6], where it exhibited a strong cytotoxic effect in several leukemia and lymphoma cell lines (such as HL60, HPBALL, Jurkat, K562, KOPTK1, MOLT4, SUPB15, and Ramos) [6,7]. In subsequent studies, we showed that bromoxib activates the intrinsic mitochondrial death pathway, since bromoxib-induced apoptosis was blocked in Jurkat cells deficient for caspase-9 (as central initiator caspase of the mitochondrial death pathway) [7]. In addition, our findings indicated that bromoxib functions as an uncoupler of the electron transport chain, exhibiting kinetics comparable to the protonophore CCCP in terms of the rapid breakdown (within 1–2 min) of the mitochondrial membrane potential (ΔΨm) and subsequent mitochondrial fragmentation. Moreover, bromoxib strongly abolished ATP production by cytosolic glycolysis and mitochondrial oxidative phosphorylation (OXPHOS). Inhibition of OXPHOS by bromoxib was mediated by targeting electron transport chain (ETC) complexes II, III, and the F_1_F_O_ ATP-synthase (complex V) [7].

In the present study, we used a mass spectrometric thermal proteome profiling (TPP) approach and noticed a rapid decrease in the abundance of 19 nucleoporins (NUPs) in TPP after 30 min of bromoxib treatment. The nuclear pore complex (NPC) is a multiprotein structure embedded in the nuclear membrane, facilitating the selective bidirectional transport of macromolecules (such as transcription factors and mRNA) across the nuclear membrane. The NPC controls the nuclear import and export, which are essential for cellular homeostasis, gene expression, signal transduction, and cell cycle progression [8]. It represents one of the largest macromolecular protein assemblies, consisting of approximately a thousand protein subunits, and has a molecular mass of 110 MDa [9,10]. The structure and function of the NPC is highly conserved from yeast to mammals and is constituted by multiple copies of 30 different NUPs, which are arranged in an octagonal symmetry around their axis [11]. The NUPs can be categorized into six different functional groups: (i) cytoplasmic filament NUPs, (ii) coat NUPs, (iii) pore membrane NUPs, (iv) adapter NUPs, (v) channel NUPs, and (vi) nuclear basket NUPs [12,13,14]. The various NUPs share homologous similarities, including the presence of β-propeller, α-helical regions, and phenylalanine and glycine-enriched (FG) repeat domains [15]. The FG repeats serve to create a diffusion barrier for small molecules (~40 kDa) and a docking site for transport receptors of larger proteins [13,16]. Transport receptors of the karyopherin superfamily transfer the majority of proteins across the NPC. Depending on the direction in which karyopherins transport their cargo proteins, they are termed importins or exportins [17]. Proteins displaying a nuclear localization signal (NLS) are bound by the adaptor protein importin α, which subsequently binds with the actual transport receptor importin β. The importin–cargo complex translocates through the NPC by interacting with a subset of NUPs. Likewise, proteins that contain a nuclear export signal (NES) are channeled through the NPC into the cytosol [18,19]. A common consequence of disrupting the functionality or structure of the NPC is cellular degeneration [20]. On the other hand, the overexpression of various nucleoporins, particularly those involved in mRNA transport, has been associated with cancer development [21,22]. Hence, dysfunction in NPC components has been implicated in several diseases, including cancer and neurodegenerative disorders [19,23,24].

Here, we show that bromoxib induced a rapid downregulation of 19 NUPs after 30 min. This apparently affected the functionality of the NPC, as evidenced by the bromoxib-mediated inhibition of the nuclear translocation and subsequent gene reporter activity of transcription factors such as NFAT and NF-κB. In addition, bromoxib inhibited the nuclear export of HIV-Tat mRNA and the subsequent import of the HIV-Tat protein into the nucleus, as determined by the decrease in Tat-dependent gene reporter luciferase activity. Inhibition of nuclear mRNA-export also affected expression of the short-lived anti-apoptotic Bcl-2 protein Mcl-1, which has been shown to induce apoptosis [25]. Thus, its ability to target both mitochondrial metabolism [7] and the NPC renders bromoxib a promising anticancer agent.

## 2. Results

### 2.1. Bromoxib Decreases the Levels of a Subset of Nucleoporins (NUPs) as Determined by Thermal Proteome Profiling (TPP)

**Figure 2:**
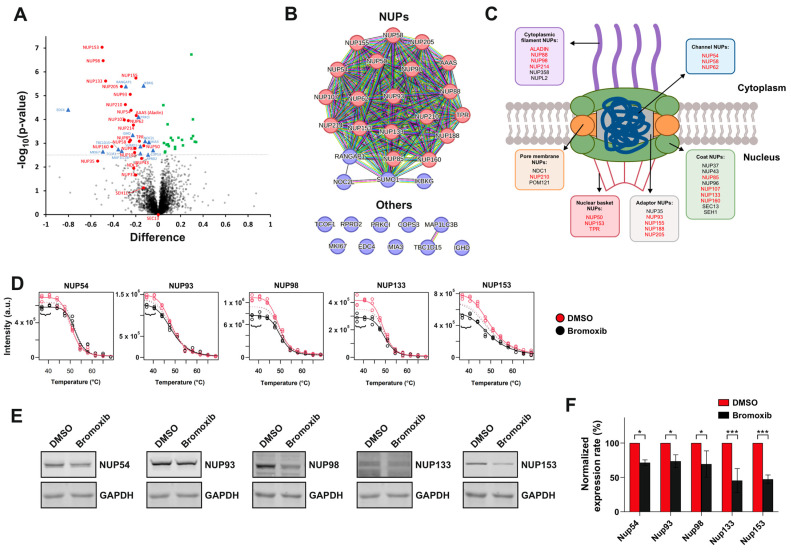
Mass spectrometric thermal proteome profiling (TPP) and immunoblot analysis of apparently downregulated selected NUPs. Mass spectrometry-based TPP data (the two lowest treatment temperatures with negligible melting effect, three biological replicates; see panel (**D**)) was used to analyze the abundance changes of proteins upon treatment of Ramos B-cell lymphoma cells with 40 µM bromoxib for a duration of 30 min versus diluent control (DMSO, 0.4% *v*/*v*). (**A**) Volcano plot of the statistical significance (expressed as the negative decadic logarithm of the Student’s *t*-test *p*-value, −log_10_(*p*-value)) versus the bromoxib-mediated abundance difference on the log_2_ scale (difference of the average log_2_ intensities of bromoxib-treated samples versus diluent controls) for each protein. Proteins with significant abundance decrease (cutoffs: -log_10_(*p*-value) > 2.5 and difference < 0) were selected for functional protein association analysis shown in panel (**B**). NUPs are labeled in red and other proteins above the cutoff in blue (difference < 0, abundance decrease) or green (difference > 0, abundance increase). The dashed line indicates the *y*-axis cutoff at -log_10_(*p*-value) > 2.5. (**B**) A functional protein association network (based on a STRING database enrichment analysis, https://string-db.org, v12.0; accessed on 06.Dec.2024) of selected (cutoffs: -log_10_(*p*-value) > 2.5 and difference < 0) proteins from panel (**A**). NUPs are labeled in red and other proteins in blue. (**C**) Schematic diagram showing the structure of the nuclear pore complex (NPC), with NUPs exhibiting a significant abundance decrease (cutoffs: -log_10_(*p*-value) > 2.5 and difference < 0) depicted in red. Scheme adapted from [13,14]. (**D**) Five selected NUP proteins (NUP54, NUP93, NUP98, NUP133, and NUP153) are shown with their respective TPP melting curves (solid lines showing the major abundance change: differential abundances were fitted using a fixed melting point for bromoxib treatments and diluent controls, dashed lines showing only minor melting point shifts; differential melting points were fitted using fixed abundance for bromoxib treatments and diluent controls). Significant abundance decrease (cutoffs: -log_10_(*p*-value) > 2.5 and difference < 0) of these proteins was observed after treatment of Ramos cells with bromoxib (40 µM bromoxib for 30 min). The curved brackets indicate the range used for the differential abundance analysis shown in panel (**A**). (**E**) Exemplary immunoblots of selected NUPs from Ramos cells treated with 40 µM bromoxib at 37 °C (employing one of the three independent biological replicates used from the TPP). GAPDH (glyceraldehyde 3-phosphate dehydrogenase) served as loading control. (**F**) Quantitative analysis of immunoblots (see panel (**B**) for representative immunoblots) of the selected NUPs. The immunoblots of NUP54, NUP93, NUP98, NUP133, and NUP153 from three independent biological replicates (n = 3) were quantified. The normalized signal intensity is shown for DMSO in red and bromoxib in black. Error bars = mean ± SD of three independent biological replicate experiments (TPP 37 °C treatment temperature). Statistical analysis was carried out using two-way ANOVA, with significance levels of * *p* ≤ 0.05; *** *p* ≤ 0.001.

We have previously applied a mass spectrometric thermal proteome profiling (TPP) approach to discover potential targets of bromoxib. This method detects drug–protein interactions in living cells by identifying proteins whose thermal stability is increased upon drug binding. Thus, we have identified a protein cluster associated with fatty acid and lipid metabolism [7]. Here, we focused on analyzing protein abundance alterations (e.g., up- or downregulation) rather than thermal protein stabilization in this TPP dataset and observed a rapid decrease in the abundance (i.e., downregulation) of several nucleoporins (NUPs) in bromoxib-treated Ramos Burkitt lymphoma cells within 30 min. As shown in the volcano plot and STRING database enrichment analysis in Figure 2A,B and Table 1, bromoxib mediated the rapid downregulation of 19 NUPs. As depicted in Figure 2C, this affected cytosolic filament-NUPs (ALADIN, NUP88, NUP98, NUP214), channel-NUPs (NUP54, NUP58, NUP62), pore membrane NUPs (NUP210), nuclear basket NUPs (NUP50, NUP153, TPR), adaptor NUPs (NUP93, NUP155, NUP188, NUP205), and coat NUPs (NUP85, NUP107, NUP133, NUP160). Since this effect was already observed at 37 °C in the melting curves of the quantitative MS-based TPP of the respective NUPs (Figure 2D), we verified by immunoblotting that bromoxib mediated the apparent downregulation of the candidate proteins NUP54, NUP93, NUP98, NUP133, and NUP153 at 37 °C. As shown in Figure 2E,F bromoxib induced a profound downregulation of all investigated NUPs at 37 °C.

Since bromoxib reduced NUP protein levels within 30 min, we presumed that this was mediated by protein aggregation rather than proteasomal degradation or transcriptional repression. To prove this, we dissolved the pellets of NP40 lysates in Laemmli sample buffer and were able to show that bromoxib apparently induced the aggregation of NUP proteins (NUP54, NUP93, NUP98, and NUP133; Supplemental Figure S2).

### 2.2. Bromoxib-Mediated Downregulation of NUPs Does Not Induce Permeability of the Nuclear Membrane

**Figure 3:**
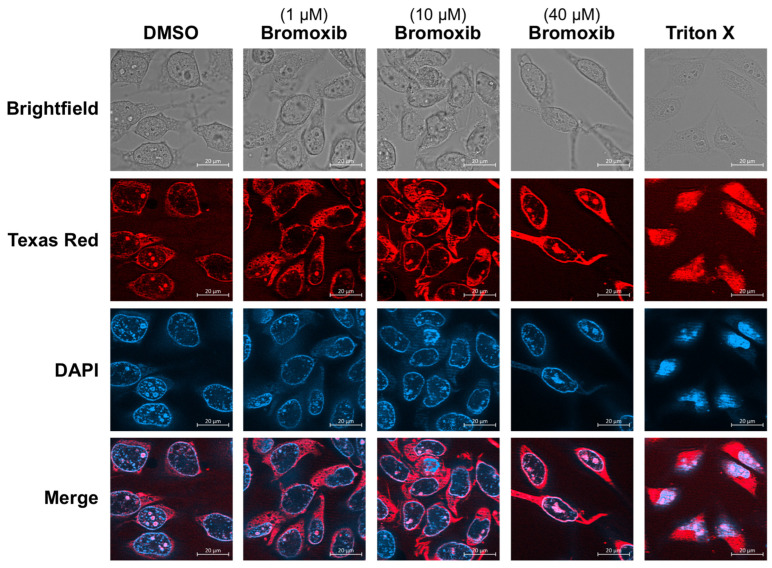
Bromoxib-mediated downregulation of NUPs does not induce nuclear permeability. HeLa cells were treated with DMSO (0.1% *v*/*v*; diluent control), 0.1 µM, 10 µM, or 40 µM bromoxib for 24 h. Subsequently, cells were treated with digitonin to permeabilize the plasma membrane (but not the nuclear membrane). Texas red dextran was then added, which can enter the cytosol (due to digitonin-mediated permeabilization of the plasma membrane) but not the nucleus. To induce nuclear membrane permeability, Triton X was used as a positive control (0.2%, 15 min incubation). Exemplary microscopy images are shown (red: Texas red dextran; blue: DAPI stained nuclei).

The decrease in the amount of NUP proteins prompted us to investigate in how far the barrier and transport function of the NPC might be affected. A possible consequence of the downregulation of numerous NUPs could be the disintegration of the NPC and the subsequent loss of its barrier function. To analyze whether bromoxib induces the permeability of NPCs, we applied the method of dextran staining of digitonin-permeabilized cells [26]. For this, HeLa cells were treated with digitonin, a detergent that permeabilizes the plasma membrane but not the nuclear membrane. Texas red dextran was then added, which can enter the cytosol (due to digitonin-mediated permeabilization of the plasma membrane) but, with a molecular weight of 70 kDa, cannot freely translocate to the nucleus. However, if the nuclear membrane is permeabilized, Texas red dextran can also enter and stain the nucleus [26]. Using this procedure, we were able to show that bromoxib did not mediate the entry of Texas red dextran into the nucleus, in contrast to Triton X-100, which was used as a positive control (Figure 3). Thus, the selectivity barrier of the NPC does not seem to be compromised upon bromoxib treatment.

### 2.3. Bromoxib Inhibits the Nuclear Import of the Transcription Factor NFAT and Subsequent NFAT Reporter Gene Activity

To investigate whether the measured decrease in the amount of NUPs affects the selective bidirectional transport of macromolecules across the NPC, we monitored the nuclear translocation of the transcription factors, NFAT, NF-κB, as well as HIV-Tat.

**Figure 4:**
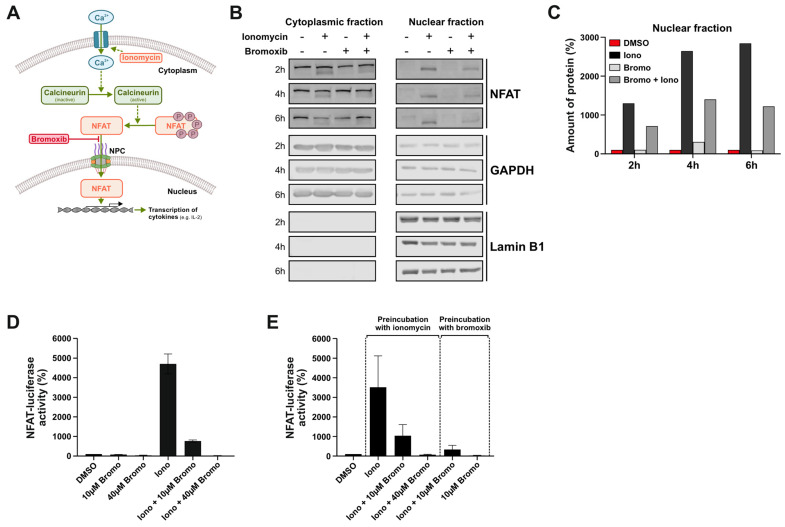
Bromoxib inhibits the nuclear import and the gene reporter luciferase activity of the transcription factor NFAT. (**A**) Overview scheme of NFAT activation. The transcription factor NFAT plays a crucial role in T cell activation and is activated upon an increase in intracellular Ca^2+^. This could be induced upon T cell receptor triggering but could also be mimicked by the Ca^2+^-ionophore ionomycin. In the cytosol, Ca^2+^ binds to calmodulin and the Ca^2+^/calmodulin complex then activates the phosphatase calcineurin. Activated calcineurin then binds and dephosphorylates NFAT, thereby revealing the nuclear localization signal (NLS) of NFAT. NFAT then translocates across the NPC to the nucleus, where it induces the expression of cytokines (such as interleukins (IL-2,-3,-4,-5,-8,-13), INFγ, GM-CSF, or TNFα). (**B**) Ramos cells were treated with the Ca^2+^-ionophore ionomycin (5 µM) or the combination of 5 µM ionomycin and 10 µM bromoxib in a kinetic for 2 h, 4 h, or 6 h. Subsequently, the cellular and nuclear fractions were prepared and subjected to immunoblotting for NFAT, GAPDH (control for cytosolic fraction), or lamin B1 (control for nuclear fraction). (**C**) Quantitative analysis of immunoblots (see (**B**) for representative immunoblots). (**D**) Jurkat cells stably expressing an NFAT-luciferase reporter gene construct were treated with 10 µM or 40 µM bromoxib, 10 µM ionomycin, or simultaneously with the combination of bromoxib and ionomycin for 6 h. The respective NFAT-luciferase activity from three independent experiments performed in triplicates is shown. (**E**) Jurkat cells stably expressing an NFAT-luciferase construct were pretreated with 10 µM ionomycin for 30 min before adding 10 µM or 40 µM bromoxib. Alternatively, Jurkat cells were pretreated with 10 µM or 40 µM bromoxib for 30 min before adding 10 µM ionomycin as indicated. The respective NFAT-luciferase activity from three independent experiments performed in triplicates was evaluated after 6 h.

The transcription factor nuclear factor of activated T cells (NFAT ) plays a crucial role in T cell activation and is activated upon an increase in intracellular Ca^2+^. This can be induced, e.g., upon triggering of the T cell receptor in T cells, but can also be mimicked by the Ca^2+^-ionophore ionomycin. In the cytosol, Ca^2+^ binds to calmodulin and the Ca^2+^/calmodulin complex, then activates the phosphatase calcineurin. Activated calcineurin binds and dephosphorylates NFAT, thereby exposing the nuclear localization signal (NLS) of NFAT. NFAT then translocates to the nucleus, where it induces the expression of cytokines (such as interleukins (IL-2,-3,-4,-5,-8,-13), INFγ, GM-CSF, or TNFα) [27]. A graphical scheme of calcineurin and NFAT signaling is provided in Figure 4A. To investigate whether the bromoxib-induced apparent downregulation of NUPs affects the nuclear translocation of NFAT, Ramos B-cell lymphoma cells were treated with ionomycin alone or the combination of ionomycin and bromoxib in a kinetic for 2 h, 4 h, or 6 h. Subsequently, the cellular and nuclear fractions were prepared and subjected to immunoblotting for NFAT. As shown in Figure 4B,C, ionomycin induced a sustained nuclear translocation of NFAT within 2–6 h. Bromoxib treatment alone had no effect on this process, but it substantially reduced the ionomycin-induced nuclear translocation of NFAT. To monitor the effect of bromoxib on the functional activity of NFAT, we used Jurkat cells stably expressing an NFAT luciferase reporter gene construct. When administered simultaneously, 10 µM bromoxib substantially reduced the ionomycin-induced NFAT luciferase activity and completely blocked it at 40 µM (Figure 4D). As shown in Figure 4E, the preincubation with 10 µM bromoxib for 30 min prior to ionomycin treatment further reduced the NFAT luciferase activity. When Jurkat cells were first treated with ionomycin for 30 min prior to the application of 10 µM bromoxib, NFAT-driven luciferase activity was also profoundly reduced, and to a similar extent compared to the preincubation (Figure E) or concurrent administration of bromoxib (Figure 4D). Thus, bromoxib appears to exert a rapid and sustained reduction on ionomycin-induced NFAT luciferase activity.

### 2.4. Bromoxib Inhibits NF-κB-Dependent Reporter Gene Activity

**Figure 5:**
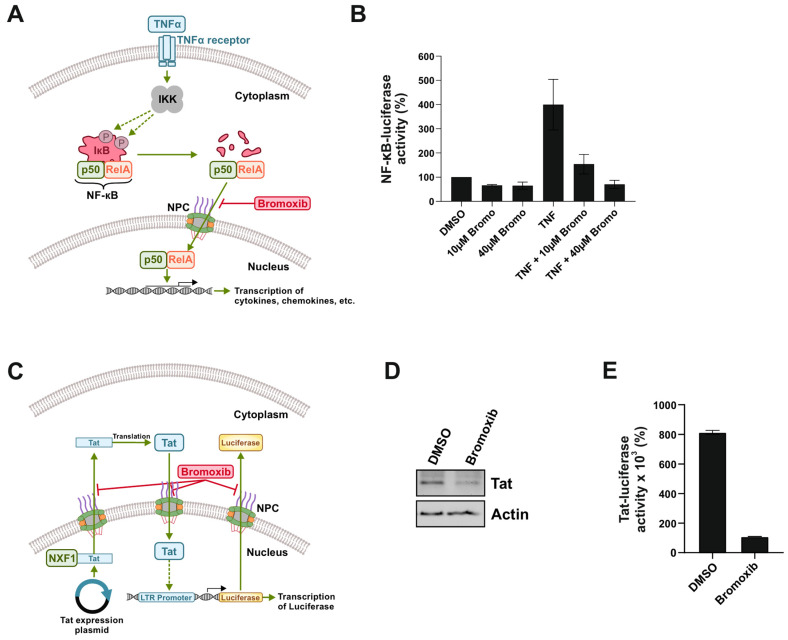
Bromoxib inhibits the NF-κB gene reporter activity, reduces the nuclear export of Tat mRNA, and inhibits subsequent Tat-driven gene reporter activity. (**A**) Schematic diagram of NF-κB activation. In the classical (canonical) signaling pathway, NF-κB is activated by proinflammatory cytokines such as TNFα. Triggering of the TNFα receptor (TNF-R1) activates the IκB kinase (IKK) complex that phosphorylates the inhibitor of NF-κB (IκB) at two specific N-terminal serine residues. The subsequent polyubiquitination and proteasomal degradation of the IκB protein leads to the release of NF-κB (p50/RelA), which then translocates across the NPC to the nucleus to induce the expression of respective target genes (such as cytokines, chemokines, growth factors, cell adhesion molecules, regulators of apoptosis, or acute phase proteins). (**B**) HEK293 cells were transiently transfected with an NF-κB luciferase reporter gene construct together with a β-galactosidase plasmid and treated with 10 ng/mL TNFα with or without 10 µM or 40 µM bromoxib for 6 h. The luciferase activity was measured and normalized to β-galactosidase activity. The respective NF-κB-dependent luciferase activity from three independent experiments performed in triplicates is shown. (**C**) Schematic overview of the HIV-Tat system used. In this system, TZM-bl cells that stably express luciferase under a Tat-dependent LTR promotor are transiently transfected with an HIV-Tat expression plasmid. The transcribed nuclear HIV-Tat mRNA is subsequently transported across the NPC into the cytosol and translated into the HIV-Tat protein. The HIV-Tat protein then translocates across the NPC to the nucleus where it induces the expression of luciferase under the HIV-1 derived LTR promoter. (**D**) TZM-bl cells were incubated with 10 µM of the pan-caspase inhibitor QVD together with 18.6 µM bromoxib or 0.4% *w*/*v* DMSO (diluent control) for 16 h. Subsequently, immunoblots for Tat or actin (loading control) were performed. (**E**) TZM-bl cells transiently transfected with HIV-Tat were incubated with 10 µM QVD together with 18.6 µM bromoxib or 0.4% *w*/*v* DMSO (diluent control) for 16 h. The respective luciferase activity from three independent experiments performed in quadruplicate is shown.

In addition to NFAT, we also examined the effect of bromoxib on the transcription factor nuclear factor κB (NF-κB). NF-κB is a ubiquitous transcription factor that plays an essential role in the regulation of a variety of biological processes such as inflammation, immune response, apoptosis, and cell proliferation. NF-κB binds as a hetero- or homodimer to specific κB-binding sites within promoters of its target genes. In unstimulated cells, NF-κB is present in the cytoplasm as an inactive complex bound to its inhibitor IκB. Numerous stimuli lead to the activation of NF-κB. In the classical (canonical) signaling pathway (Figure 5A), NF-κB can be activated by proinflammatory cytokines such as TNFα, IL-1, or toll-like receptor (TLR) ligands (e.g., lipopolysaccharide (LPS)). Upon stimulation, the IκB kinase (IKK) complex is activated and phosphorylates the IκB protein at two specific N-terminal serine residues. The subsequent polyubiquitination and proteasomal degradation of the IκB protein leads to the release of NF-κB, which then translocates to the nucleus to induce the expression of respective target genes (such as cytokines, chemokines, growth factors, cell adhesion molecules, regulators of apoptosis, or acute phase proteins) [28]. To investigate the effect of bromoxib on NF-κB activation, HEK293 cells were transiently transfected with an NF-κB-controlled luciferase reporter gene construct and treated with TNFα with or without bromoxib. As shown in Figure 5B, 10 µM of bromoxib strongly reduced and 40 µM of bromoxib completely abolished the TNFα-induced NF-κB luciferase activity. This observation supports the idea that bromoxib interferes with the import of NF-κB by disrupting NPC function.

### 2.5. Bromoxib Reduces the Nuclear Export of Tat mRNA, and Inhibits Subsequent Tat-Driven Reporter Gene Activity

To monitor the nuclear mRNA-export and subsequent protein-import, we made use of HIV-Tat and Tat-dependent luciferase activity in TZM-bl cells. The transactivator of transcription (Tat) of the human immunodeficiency virus (HIV) promotes the efficient viral replication by stabilization of the transcription of viral genes. The HIV-1 replication starts by the binding of the cellular transcription factors NF-κB, NFAT, and Sp1 to the long terminal repeat (LTR) region of the HIV-1 promoter, where RNA polymerase II subsequently binds and starts viral transcription. The HIV-1 transcript is then produced and the fully spliced HIV-1 mRNA transported into the cytoplasm in order to be translated into the HIV-1 protein Tat [29]. The transcription rate of the HIV-1 genome is drastically increased when Tat enters the nucleus and binds to the 5′ end of the transactivation response element (TAR) hairpin, a cis-acting RNA regulatory element present in the newly synthesized HIV-1 mRNA transcript [29]. We made use of this system by transiently transfecting HIV-Tat into TZM-bl cells that stably express luciferase under a Tat-dependent LTR promotor. The transcribed nuclear HIV-Tat mRNA is subsequently transported across the NPC into the cytosol and translated into the HIV-Tat protein. The HIV-Tat protein then translocates to the nucleus, where it induces the expression of luciferase under the HIV-1 derived LTR promoter (Figure 5C). Thus, this system enables the monitoring of the nuclear mRNA export of HIV-Tat as well as the subsequent entry of the HIV-Tat protein via luciferase activity. In order to avoid potential bromoxib-mediated protein degradation by caspases after 24 h of bromoxib treatment, the pan-caspase inhibitor QVD was applied. As shown in Figure 5D, treatment with bromoxib reduced the protein expression of HIV-Tat, which was most likely attributed to the inhibition of mRNA export of HIV-Tat. Although HIV-Tat protein was still detectable, bromoxib completely abrogated HIV-Tat luciferase activity (Figure 5E), indicating the inhibition of the nuclear import of the HIV-Tat protein.

### 2.6. Bromoxib Inhibits the Expression of Short-Lived Proteins Such as the Anti-Apoptotic Bcl-2 Protein Mcl-1

**Figure 6:**
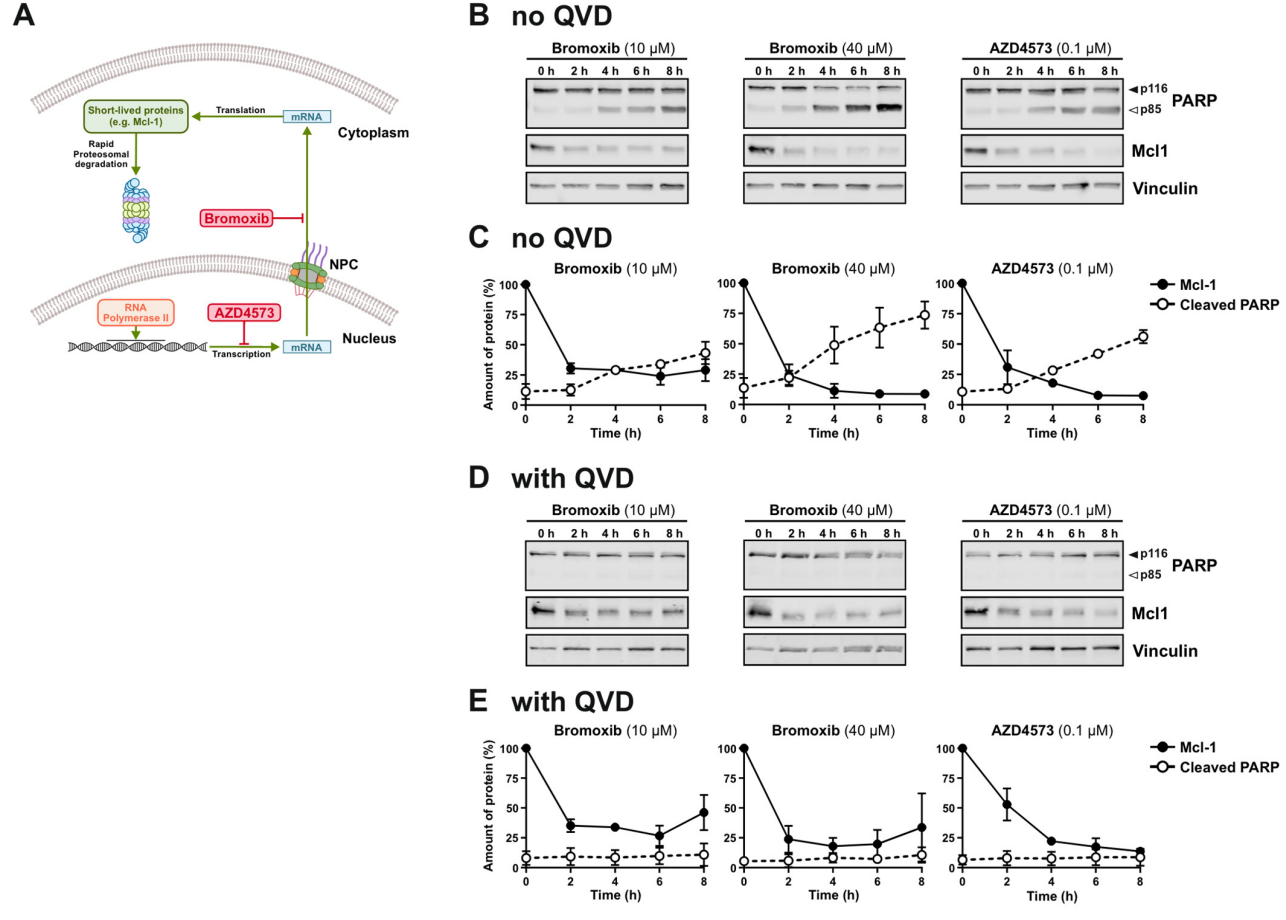
Bromoxib inhibits the expression of the short-lived anti-apoptotic Bcl-2 protein Mcl-1. (**A**) Schematic illustration of the effect of RNA polymerase II inhibition by the CDK9 inhibitor AZD4573 on the subsequent protein expression of short-lived proteins such as Mcl-1. RNA polymerase II mediates the transcription of mRNA in the nucleus, which then translocates across the NPC into the cytosol. There, mRNA is translated at the ribosomes into respective proteins. Finally, proteins are degraded by the proteasome. One of the short-lived proteins is the anti-apoptotic Bcl-2 protein Mcl-1. Studies by Cidado et al. have shown that the selective CDK9-inhibitor AZD4573 inhibits the CDK9-mediated activation of RNA polymerase II and induces the downregulation of Mcl-1 mRNA, followed by a rapid downregulation of Mcl-1 at the protein level [25]. (**B**) Ramos cells were treated with 10 µM or 40 µM of bromoxib, or 0.1 µM of the CDK9 inhibitor AZD4573 in a kinetic for 0 h, 2 h, 4 h, 6 h or 8 h. Treatment with 0.2% *w*/*v* DMSO for 8 h served as diluent control. Subsequently, immunoblots for the anti-apoptotic Bcl-2 protein Mcl-1, the caspase substrate poly(ADP-ribose) polymerase-1 (PARP), or vinculin (loading control) were performed. Solid arrowheads indicate the uncleaved form of PARP (p116); open arrowheads indicate the cleaved form (p85). (**C**) Quantitative analysis of immunoblots (see (**B**) for representative immunoblots) from three independent biological replicates (n = 3). (**D**) To avoid caspase-mediated protein degradation, Ramos cells were pretreated with 10 µM of the caspase-inhibitor QVD for 30 min. Subsequently, cells were treated as described in (**B**). (**E**) Quantitative analysis of immunoblots (see (**D**) for representative immunoblots) from three independent biological replicates (n = 3).

The former experiments indicate that bromoxib inhibits not only the nuclear import of proteins (such as NFAT, NF-κB and HIV-Tat) but also the nuclear export of HIV-Tat mRNA. If this is the case, then bromoxib should also affect the expression of short-lived proteins such as the anti-apoptotic Bcl-2 protein Mcl-1. Studies by Cidado et al. have shown that the selective CDK9-inhibitor AZD4573 inhibits the CDK9-mediated activation of RNA polymerase II and induces the downregulation of Mcl-1 mRNA, followed by a rapid downregulation of Mcl-1 at the protein level [25] (Figure 6A). Therefore, we investigated whether bromoxib inhibits the expression of Mcl-1. For this, we treated Ramos cells with bromoxib for up to 8 h and immunoblotted for Mcl-1 and the caspase substrate poly(ADP-ribose) polymerase-1 (PARP). As shown in Figure 6B,C, bromoxib inhibited the expression of Mcl-1 in similar rapid kinetics as the CDK9 inhibitor AZD4573. A pronounced cleavage of the caspase substrate PARP was observed as early as 4 h upon treatment with bromoxib or AZD4573. To avoid additional protein degradation by caspases, the pan-caspase inhibitor QVD was added. However, QVD did not affect the degradation of the short-lived Mcl-1 protein but, as expected, inhibited the proteolytic cleavage of the caspase substrate PARP (Figure 6D,E).

## 3. Discussion

Shuttling of proteins across the nuclear membrane is essential for the regulation of the cell cycle and proliferation of both normal and malignant cells. Consequently, dysregulation of this fundamental process affects crucial cellular processes such as tumor growth, inflammatory response, cell cycle, and apoptosis. In this context, NPCs exert a central role in the regulation of transport-dependent gene expression and thereby affect cancer development and progression [19,23,24]. The nucleo-cytoplasmic transport across NPCs regulates the intracellular localization of tumor suppressors and oncoproteins, which is frequently deregulated in cancer. Accordingly, abnormal localization of tumor suppressors and oncoproteins results in their respective inactivation or overactivation. [19,23,24]. In this context, it was shown that inhibition of NPC formation induces selective cytotoxicity in cancer cells and prevents tumor growth [30]. NUP107 (also downregulated by bromoxib; Figure 2 and Table 1) has been identified as a potent oncogene in glioblastoma and reduced NUP107 expression leads to the stabilization of the tumor suppressor p53 in the nucleus. Another NUP downregulated by bromoxib is NUP153, which is responsible for p53 degradation by tethering proteasomes [19,31]. TPR (also downregulated by bromoxib) controls the total number of NPCs per nucleus, which can result in increased nucleo-cytoplasmic transport capacity, providing multidrug resistance, as cells with more NUPs are more capable of exporting drugs from the nuclei [19]. In addition, elevated levels of other NUPs downregulated by bromoxib in TPP (i.e., NUP188, NUP210, NUP85, NUP62, and NUP214) were identified in metastatic prostate cancer, suggesting that NPC dysregulation may drive aggressive cancer phenotypes [19,32,33,34].

Consequently, targeting of nuclear import and export has emerged as a potential strategy for cancer treatment. Among the various targets involved in nucleo-cytoplasmic transport, the nuclear export receptor XPO1 (exportin 1, CRM1) represents the most advanced therapeutic target for cancer therapy due to its hyperactivity in various malignant tumors, such as ovarian cancer, glioma, and osteosarcoma, as well as pancreatic cancer and cervical cancer [19,23,24]. XPO1 plays a fundamental role in regulating the nucleo-cytoplasmic transport by exporting proteins containing nuclear export signals (NES), including numerous oncogenes and tumor suppressors. XPO1 accomplishes this by interacting with nucleoporins, including NUP214 and NUP88, which are essential for docking and translocation of cargo across the NPC. Dysregulation of XPO1 perturbs the delicate equilibrium between oncogenes and tumor suppressors, thereby promoting aberrant cellular proliferation and tumorigenesis [19,23,24]. There exist a number of XPO1 inhibitors, such as leptomycin B, ratjadones, PKF050-638, valtrate, ACA, BS9106, selinexor/KPT-330, and verdinexor/KPT-335. Selinexor and verdinexor are highly potent and orally bioavailable. KPT-330 is currently the only agent undergoing phase I/II clinical trials for hematological and solid malignancies [23]. Beside these XPO1 inhibitors, other strategies include antibodies targeting the nuclear export signal and altering the structure of the NPC [19].

In previous studies, we have shown that the polybrominated diphenyl ether bromoxib displays a strong cytotoxic effect in several leukemia and lymphoma cell lines (such as HL60, HPBALL, Jurkat, K562, KOPTK1, MOLT4, SUPB15, and Ramos), but also in solid tumor cell lines (such as glioblastoma cell lines SJ-GBM2 and TP365MG) [4,6,7]. In addition, bromoxib had a 3.2-fold lower IC_50_ value in primary leukemia cells from patients with acute myeloid leukemia (AML) compared to peripheral blood mononuclear cells (PBMNC) from healthy donors, providing a therapeutic window [4,6]. We also showed that bromoxib activated the intrinsic mitochondrial apoptosis pathway, disrupted the mitochondrial membrane potential (ΔΨm) within 1–2 min, and induced mitochondrial fragmentation. Moreover, bromoxib functioned as an uncoupler of the mitochondrial electron transport chain and inhibited ATP production by cytosolic glycolysis and mitochondrial OXPHOS. The latter was mediated by targeting electron transport chain complexes II and III and the F_1_F_O_ ATP-synthase (complex V) [7]. In the same study, using a mass spectrometric TPP approach, we observed the bromoxib-mediated thermal stabilization of a protein cluster associated with fatty acid and lipid metabolism, including ECH1, ACADVL, ACSL4, and HADHA/B, implicating these proteins as potential targets of bromoxib. However, since bromoxib did not inhibit fatty acid oxidation, but rather increased it, we excluded a possible role of proteins involved in fatty acid and lipid metabolism as direct targets of bromoxib [7].

In this study, using the mass spectrometric TPP dataset from the previous study, we focused on analyzing protein abundance changes (e.g., up- or downregulation) rather than thermal protein stabilization and were able to show that bromoxib has the additional potential to induce the rapid downregulation of 19 NUPs (Figure 2 and Table 1). Thus, we observed a reduced amount of NUPs in cell extracts of NP40-soluble membrane proteins obtained during TPP sample preparation. The bromoxib-mediated downregulation of NUPs occurred within 30 min and was most likely due to aggregation of NUP proteins (Supplemental Figure S2) rather than inhibition of their protein expression. This obviously affected the doorkeeper function of the NPC, as bromoxib obstructed the nuclear protein-import of the transcription factors NFAT (Figure 4), NF-κB (Figure 5A,B), and HIV-Tat, as well as the export of HIV-Tat mRNA (Figure 5C–E).

Accordingly, inhibition of the nuclear mRNA-export affects the subsequent protein expression, especially of short-lived proteins. In this context, we showed that bromoxib inhibited the expression of the short-lived anti-apoptotic Bcl-2 protein Mcl-1 with similar kinetics as the selective CDK9-inhibitor AZD4573 (Figure 6), which inhibits transcription by targeting RNA polymerase II. In this regard, Cidado et al. showed that AZD4573 induced a rapid downregulation of Mcl-1 mRNA, followed by the downregulation of Mcl-1 on protein level within 4 h, which preceded the onset of subsequent caspase activation in the AML cell line MV-4-11 [25]. As a result, downregulated Mcl-1 could no longer neutralize the pro-apoptotic Bcl-2 protein Bak, which subsequently induced apoptosis. Accordingly, AZD4573-mediated apoptosis was abolished in Bak knockdown cells [25].

However, the extent to which (i) the bromoxib-mediated targeting of the NPC and subsequent inhibition of Mcl-1 expression or (ii) targeting of the mitochondrial metabolism by the rapid breakdown of the mitochondrial membrane potential (ΔΨm) and OXPHOS inhibition are responsible for the activation of the mitochondrial apoptosis pathway remains to be determined. Nevertheless, bromoxib’s ability to target both NPCs and mitochondrial metabolism makes it a promising anticancer drug.

## 4. Materials and Methods

### 4.1. Reagents

Bromoxib (4,5,6-tribromo-2-(2′,4′-dibromophenoxy) phenol, formerly termed P01F08 [4,6]) was obtained from the biobank (natural compound library) of the Institute for Pharmaceutical Biology and Biotechnology of the Heinrich Heine University Duesseldorf. Bromoxib was freshly prepared and dissolved in DMSO at 10 mM stock solution. Until use for the assays, the compound was kept at −20 °C in a temperature-controlled refrigerator. Ionomycin (#10634) was obtained from Sigma (Munich, Germany). The pan-caspase inhibitor N-(2-Quinolyl)valyl-aspartyl-(2,6-difluorophenoxy)methyl ketone (Q-VD-OPh, QVD, #S7311) and the selective CDK9-inhibitor AZD4573 (S8719) were obtained from Selleckchem (Houston, TX, USA). All other reagents for which a manufacturer is not explicitly specified were obtained from Carl Roth (Karlsruhe, Germany).

### 4.2. Cell Lines and Cell Culture

Ramos cells (human B cell Burkitt lymphoma) were kindly provided by Michael Engelke (Institute of Cellular and Molecular Immunology, University Hospital Goettingen, Goettingen, Germany). Jurkat cells stably transfected with a luciferase reporter gene construct controlled by the nuclear factor of activated T cells (NFAT; kindly provided by Burkhard Schraven; Institute of Molecular and Clinical Immunology, University of Magdeburg, Germany) were cultured in RPMI media with 10% FCS. HeLa cells (human cervix carcinoma; #ACC-57) and HEK293 cells (human embryonic kidney) were cultured in DMEM with 10% FCS. Additionally, 10 mM Hepes, 100 U/mL penicillin and 100 µg/mL streptomycin was added in all cell culture media, and the cells were incubated at 37 °C and 5% CO_2_ in a humidity-saturated atmosphere. TZM-bl cells were obtained through the NIH AIDS Reagent Program, Division of AIDS, NIAID, NIH from Dr. John C. Kappes, Dr. Xiaoyun Wu, and Tranzyme Inc., Durham, UK, (ARP-8129). TZM-bl cells were cultured in DMEM with 10% FCS, 100 U/mL penicillin, and 100 µg/mL streptomycin at 37 °C and 5% CO_2_ in a humidity-saturated atmosphere.

### 4.3. Transfections

To measure the transcriptional activity of NF-κB, HEK293 cells were seeded into six-well plates at a concentration of 4 × 10^5^/well and transiently transfected the following day with a NF-κB luciferase reporter plasmid containing six κB-binding sites in front of the luciferase promoter at a concentration of 800 ng/well. As an internal control for transfection efficiency a β-galactosidase (lacZ) vector was cotransfected at a concentration of 200 ng/well. The plasmids were diluted in OPTI-MEM and TurboFect transfection reagent (Thermo Scientific, Waltham, MA, USA) was added. After incubation for 30 min at room temperature, the transfection reagent/DNA mixture was added to the cells.

Transient transfection experiments with a pcDNA3.1 based HIV-1 Tat expression plasmid were performed in twelve-well plates at 1.25 × 10^5^ cells/well by using TransIT-LT1 Transfection Reagent (Mirus Bio, Madison, WI, USA, LLC US MIR2305), according to the manufacturer’s instructions. For treatment, 18.6 µM bromoxib together with 10 µM QVD was added 8 h post-transfection; 0.4% *w*/*v* DMSO was used as a diluent control. At 24 h post-transfection, cells were harvested in 2 mM EDTA in PBS for 2–5 min at room temperature, and the cell pellet was lysed in ice-cold 20–60 μL RIPA buffer (25 mM Tris-HCl (pH 7.6), 150 mM NaCl, 1% NP-40, 1% sodium deoxycholate, 0.1% SDS, protease inhibitor cocktail) (EMD Millipore, Burlington, MA, USA). After freezing at −80 °C for 10 min, samples were briefly centrifuged, and the resulting supernatant was mixed with 5× protein sample buffer (250 mM Tris-HCl pH 6.8, 2% SDS, 20% glycerol, 0.05% bromophenol blue, and 50 mM DTT) at a 4:1 ratio and heated at 95 °C for 5 min prior to analysis via immunoblot.

### 4.4. Luciferase Assays

For determination of NF-κB-dependent transcriptional activity, HEK293 cells were treated with bromoxib (10 µM or 40 µM and/or 10 ng/mL of TNF-α) one day after transient transfection with a κB-dependent luciferase reporter and a lacZ plasmid. For the NFAT experiment, total of 1 × 10^6^ cells/well of Jurkat T cell leukemia cells stably expressing NFAT luciferase were seeded and subsequently treated in accordance with the experimental design (10 µM or 40 µM bromoxib and/or 5 µM ionomycin). Following a six-hour incubation period, the cells of both experiments were harvested by centrifugation and frozen. Subsequently, cells were lysed with 100 µL of lysis buffer (comprising 25 mM Gly-Gly, 15 mM MgSO_4_, 4 mM EGTA, 1 mM DTT and 1% Triton X-100) and incubated on ice for 15 min. The samples were then centrifuged (13,000 rpm, 5 min, 4 °C), and 10 µL of the lysates was combined with 140 µL of the assay buffer (15 mM Kpi, 25 mM Gly-Gly, 15 mM MgSO_4_, 4 mM EGTA, 1 mM DTT, 2 mM ATP). In the plate reader, 100 µL of the luciferin solution (0.3 mg/mL) was added, and the resulting luciferase intensity was measured with a microplate spectrophotometer (Synergy Mix plate reader, BioTek Instruments, Winooski, VT, USA). A β-galactosidase assay was also conducted on the transfected HEK293 cells to normalize the transfection rate per sample. Using the Galacto-Light Plus system (Applied Biosystems, Thermo Scientific, Waltham, MA, USA), the activity of this enzyme could also be detected luminometrically. Subsequently, the blanks of each sample were subtracted, the NF-κB intensity was normalized to the β-galactosidase intensity, and the values were normalized in relation to the DMSO control. After transient transfection with an HIV-1 Tat expression plasmid, TZM-bl cells were treated with 18.6 µM Bromoxib together with 10 µM QVD 8 h post-transfection; DMSO was used as a control. At 24 h post-transfection, cells were harvested in 2 mM EDTA in PBS for 2–5 min at room temperature, and the cell pellet was lysed in 200 μL 1× Lysis-Juice (PJK). The firefly substrates (D-Luciferin and ATP, PJK) were dissolved in the Beetle-Juice reaction buffer (PJK), and the luciferase activity was measured in white flat-bottomed 96-well plates (Nunc™, Thermo Scientific, Waltham, MA, USA)). The measurement was performed in quadruplicate by the Tecan Infinite^®^ 200 machine and the i-control 1.12 software (Tecan Life Sciences, Maennedorf, Switzerland), where the integration time was 10,000 ms. The light emitted by the luciferase activity was measured in relative light units (RLU).

### 4.5. Immunoblotting

For the separate lysis of nucleus and cytoplasm and for the Mcl-1 immunoblots, 2 × 10^6^ cells/mL were seeded, treated, and incubated for the defined time. The cells were then harvested by centrifugation (3000 rpm, 5 min, 4 °C) and frozen at −20 °C. For the separated lysis, the cells were lysed for two minutes with 40 µL of digitonin lysis buffer (0.00625% digitonin, 20 mM HEPES pH 7.5, 100 mM sucrose, 2.5 mM MgCl_2_, 100 mM KCl, supplemented with protease inhibitors (Sigma, St. Louis, MO, USA, #P2714) and PhosStop (Sigma, 4906837001)). Following the brief lysis period, the samples were directly centrifuged (14,000 rpm, 3 min, 4 °C) to separate the cytoplasmic proteins in the supernatant from those of the cellular organelles in the pellet. Subsequently, the samples were lysed further to disrupt the nuclear membrane. The same lysis is the first step in the protein preparation of the Mcl-1 immunoblot samples. Here, 40 µL of the lysis buffer (comprising 20 mM Tris-HCl, 150 mM NaCl, 1% Triton X-100, 0.5 mM EDTA, 10 mM NaF, 2.5 mM Na_4_P_2_O_7_, PhosStop and protease inhibitor) was used. Following a 30 min incubation, a second centrifugation was performed (13,300 rpm, 15 min, 4 °C). This step facilitated the separation of proteins from cellular organelles (e.g., nucleus, mitochondria) present in the supernatant from residual cellular debris. For Figure 2E, the samples of the TPP analysis were used to perform immunoblot analysis. The protein concentration of all samples was determined by the Bradford assay and the samples were diluted in the respective lysis buffer and boiled for 5 min at 95 °C in Laemmli sample buffer. For Supplemental Figure S2, the pellets of the lysates were boiled for 5 min at 95 °C in Laemmli sample buffer. The SDS-PAGE and immunoblot procedures were conducted according to established standard workflows. The final step involved the application of target protein-specific primary antibodies, which were diluted in 1× TBS-T, supplemented with 0.05% NaN_3_ and 5% BSA, namely anti-β-Actin (Sigma, 1:5000), anti-GAPDH (glyceraldehyde 3-phosphate dehydrogenase; Abcam, Cambridge, England, #ab8245, 1:5000), anti-lamin B1 (Proteintech, Rosemont, IL, USA, #12987-1-AP, 1:1000), anti-NFAT (Becton Dickinson, Franklin Lakes, NJ, USA, #556601, 1:200), anti-Nup54 (Proteintech, #16232-1-AP, 1:500), anti-Nup93 (SantaCruz, Santa Cruz, CA, USA, #sc-374400, 1:200), anti-Nup98 (Sigma, #MABE1040, 1:1000), anti-Nup133 (Abcam, #ab155990, 1:1000), anti-Nup153 (Abcam, #ab24700, 1:500), anti-Mcl-1 (Cell Signaling Technology, Danvers, MA, USA, 94296S, 1:500), anti-PARP (Cell Signaling Technology, 1:1000), anti-Tat (Abcam, #ab43014, 1:2000), and anti-vinculin (Sigma, #V9131, 1:2000), followed by the use of fluorescence-coupled secondary antibodies (LI-COR Biosciences) for protein detection on a PVDF membrane via the LI-COR Odyssey^®^ imaging system. Immunoblots of Figure 5D were not detected using the LI-COR Odyssey^®^ imaging system, but with peroxidase-conjugated affinity-purified secondary antibodies and enhanced chemiluminescence (ECL). The density of the individual protein band was determined using Image StudioTM Lite Version 5.2. The density of each protein was normalized by that of the respective loading control (vinculin, lamin B1 or GAPDH). To determine the fold change, each normalized ratio was divided by the normalized band of the DMSO control. A fold change of 100% was therefore observed for the control. The results were visualized with GraphPad Prism 8.

### 4.6. Permeability Assays and Microscopy

Nuclear permeability of cells was analyzed according to dextran staining of digitonin permeabilized cells [26]. HeLa cells were seeded at a concentration of 0.05 × 10^6^ cells/well in 24-well plates on autoclaved coverslips. The next day, cells were treated with bromoxib or DMSO for 24 h or with 0.2% Triton X for 15 min as a positive control. Subsequently, the cells were washed with PBS and incubated for 30 min in permeabilization buffer (comprising 15 µg/mL digitonin, 20 mM HEPES, 110 mM KOAc, 5 mM MgCl_2_, 0.25 M sucrose, and protease inhibitors). Following three washing steps with diffusion buffer (20 mM HEPES, 110 mM KOAc, 5 mM NaCl, 2 mM MgCl_2_, 0.25 M sucrose, and protease inhibitors), the cells were incubated for 10 min in diffusion buffer with DAPI (Roth, #63351, 1:500) and Texas red dextran (Thermo Fisher, 11580226, 0.5 mg/mL). The coverslips were sealed with nail polish and the microscopy was conducted on the same day. The staining was analyzed microscopically using an Axio Observer microscope from ZEISS (Jena, Germany) with a 40× oil immersion objective.

### 4.7. MS-Based Differential Proteome Analysis from Thermal Proteome Profiling (TPP) Data

Thermal proteome profiling (TPP) of bromoxib-mediated stabilization of proteins has been described in detail in Schmitt et al. [7]. Due to the special arrangement of samples for 10-plex TMT labeling, enabling the direct quantitative comparison between proteins from bromoxib-treated and control samples at each temperature and replicate, the bromoxib-mediated protein abundance could be analyzed as well from the same samples and dataset. Very similar approaches have been described previously [35,36], and we will only detail how the quantitative protein information was extracted from the existing TPP dataset [7] and used for statistics. TPP data were prepared from the TMT reporter ion intensities by a three-step normalization procedure described in step (1) of subsection “Statistical analysis of melting curves” of section “Thermal proteome profiling—temperature range (TPP-TR)” within the “Supplemental Materials and Methods” of Schmitt et al. [7]. Melting curves were explicitly not normalized to a maximum plateau of 1 separately for treatment and control, as performed for thermal protein (de)stabilization analysis as performed in Schmitt et al. [7]. Then, the log_2_ transformed intensity values at the lowest two temperatures (37 °C and 41 °C) were employed for a classical MS-based differential proteome analysis (bromoxib treatment vs. diluent control). At these temperatures, no significant melting behavior of the proteome was observed, so that initial abundance effects dominated. The data for 37 °C, DMSO, replicate 2 were excluded from the analysis (outlier in PCA analysis performed as quality control) as already performed and described in Schmitt et al. [7]), resulting in 6 datapoints for bromoxib treatment and 5 datapoints for the diluent control per protein. The difference of the average log_2_ intensities of bromoxib-treated samples versus diluent controls for each protein served to quantify bromoxib-induced protein abundance changes (“difference”, *x*-axis in Figure 2A). The statistical significance of the difference between bromoxib treatment versus diluent control was evaluated from Student’s *t*-test (two-tailed distribution, homoscedastic) *p*-values per protein displayed as negative decadic logarithm (“-log_10_(*p*-value)”, *y*-axis in Figure 2A). The cutoff was set to -log_10_(*p*-value) = 2.5, and the proteins selected as abundance decreased (difference < 0) or increased (difference > 0) were analyzed for their network linkages using STRING version 12.0 (https://string-db.org/; accessed on 6 December 2024).

### 4.8. Statistical Analyses

Error bars represent the standard deviation. If not stated otherwise, all statistical analyses were conducted using Prism v8.02 (GraphPad Software, La Jolla, CA, USA).

## Figures and Tables

**Table 1:** List of proteins with decreased abundance in thermal proteome profiling (TPP).

Protein Name	Gene Name	Difference	−log10 (*p*-Value)
Nucleoporins (NUPs)			
ALADIN	AAAS	−0.20	4.16
NUP50	NUP50, NPAP60L	−0.13	2.89
NUP54	NUP54	−0.24	4.39
NUP58	NUP58, KIAA0410, NUP45, NUPL1	−0.26	3.06
NUP62	NUP62, DKFZp547L134, FLJ20822, FLJ43869, IBSN, MGC841, p62, SNDI	−0.27	3.95
NUP85	NUP85 FLJ12549, NUP75	−0.25	3.12
NUP88	NUP88, MGC8530	−0.21	2.78
NUP93	NUP93, KIAA0095	−0.25	5.06
NUP98	NUP98, NUP196, NUP96, Nup98-96, Nup98-Nup96	−0.49	6.47
NUP107	NUP107, NUP84	−0.30	3.98
NUP133	NUP133, FLJ10814	−0.47	5.61
NUP153	NUP153, HNUP153	−0.50	7.04
NUP155	NUP155, KIAA0791, N155	−0.20	5.74
NUP160	NUP160, FLJ22583, KIAA0197	−0.41	2.86
NUP188	NUP188, KIAA0169	−0.21	2.61
NUP205	NUP205, C7orf14, KIAA0225	−0.33	5.39
NUP210	NUP210, FLJ22389, GP210, KIAA0906, POM210	−0.29	4.62
NUP214	NUP214, CAIN, CAN, D9S46E, N214	−0.22	3.76
TPR	TPR	−0.17	3.08
Other proteins			
COPS3	COPS3, CSN3, SGN3	−0.05	2.70
EDC4	EDC4, Ge-1, HEDLS, RCD-8	−0.80	4.41
IGHD	IGHD, FLJ00382, FLJ46727, MGC29633	−0.23	3.35
IKBKG	IKBKG, FIP-3, FIP3, Fip3p, IKK-gamma, IKKAP1, IKKG, IP1, IP2, NEMO, ZC2HC9	−0.13	5.42
MAP1LC3B	MAP1LC3B, ATG8F	−0.16	2.57
MIA3	MIA3, FLJ39207, KIAA0268, TANGO, TANGO1, UNQ6077	−0.08	3.05
MKI67	MKI67, Ki-67, MIB-1, PPP1R105	−0.49	2.65
NOC2L	NOC2L, DKFZP564C186, NET15, NET7, NIR, PPP1R112	−0.12	3.08
PRKCI	PRKCI, DXS1179E, PKCI	−0.18	4.10
RANGAP1	RANGAP1, Fug1, KIAA1835, SD	−0.29	5.39
RPRD2	RPRD2, FLJ32145, HSPC099, KIAA0460	−0.09	2.52
SUMO1	SUMO1, GMP1, OFC10, PIC1, SMT3C, SMT3H3, SUMO-1, UBL1	−0.16	2.87
TBC1D15	TBC1D15, DKFZp761D0223, FLJ12085	−0.36	2.75
TCOF1	TCOF1, TCS, Treacle	−0.33	2.68

Ramos cells were incubated with 40 µM bromoxib or diluent control (DMSO, 0.4% *w*/*v*) for 30 min, and proteins with decreased abundance were identified in the dataset of mass spectrometry-based thermal proteome profiling (TPP) using the cutoffs -log10(*p*-value) > 2.5 and difference < 0.

## Data Availability

The data presented in this study are available on request from the corresponding author.

## Supplementary Materials

The following supporting information can be downloaded at: https://www.mdpi.com/article/10.3390/md23030108/s1, Figure S1: ^1^H-NMR spectrum of bromoxib (4,5,6-tribromo-2-(2’,4’-dibromophenoxy) phenol); Figure S2: Bromoxib mediates the aggregation of nucleoporins (NUPs).

## Abbreviations

The following abbreviations are used in this manuscript:

bromoxib	4,5,6-tribromo-2-(2′,4′-dibromophenoxy) phenol
HIV	human immunodeficiency virus
NFAT	nuclear factor of activated T cells
NF-κB	nuclear factor κB
NPC	nuclear pore complex
NUPs	nucleoporins
PBDE	polybrominated diphenyl ether
Tat	transactivator of transcription
TPP	thermal proteome profiling
