# Supplementary material for: The Polybrominated Diphenyl Ether Bromoxib Disrupts Nuclear Import and Export by Affecting Nucleoporins of the Nuclear Pore Complex
Source: Mar Drugs. 2025 Feb 28;23(3):108. doi: 10.3390/md23030108 (PMC11943847; doi:10.3390/md23030108)
Supplement: Supplementary file 1 [file marinedrugs-23-00108-s001.zip › marinedrugs-3485334-supplementary.pdf]

Supplemental Information

Supplemental Figures

Supplemental Figure 1: ^{1}H-NMR spectrum of bromoxib (4,5,6-tribromo-2-(2′,4′-dibromophenoxy) phenol)

Supplemental Figure 1 Bromoxib mediates the aggregation of nucleoporins (NUPs)

SUPPLEMENTAL FIGURES

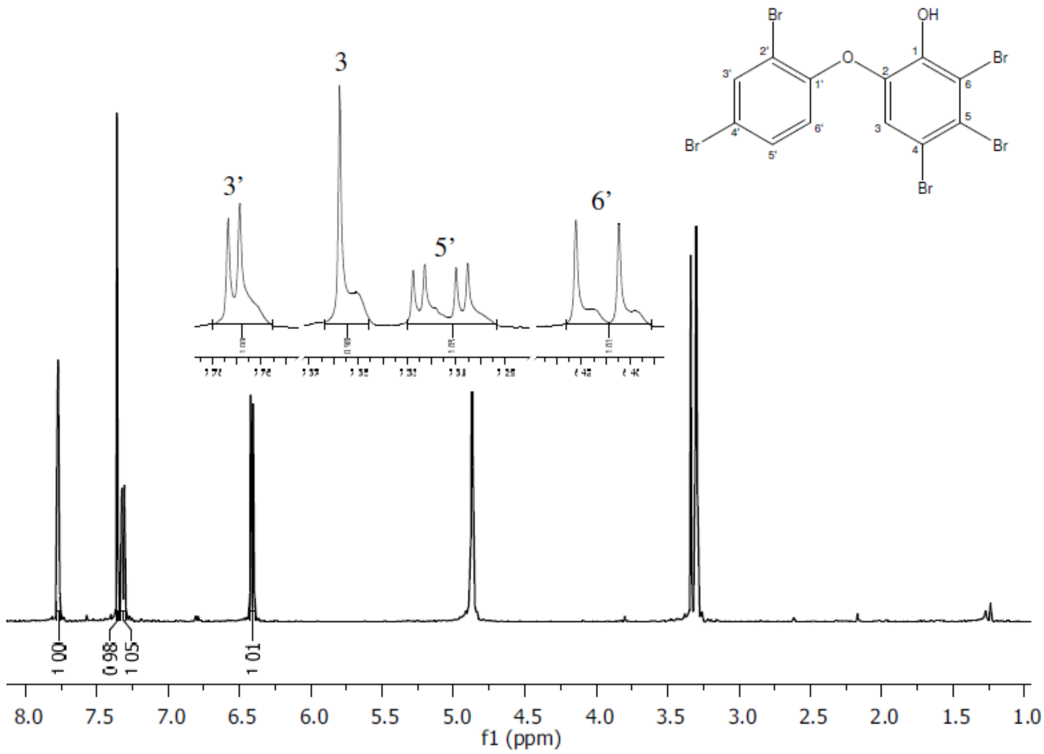

Suppl. Figure 1. ^{1}H-NMR spectrum of bromoxib (4,5,6-tribromo-2-(2′,4′-dibromophenoxy) phenol) in CD3OD.

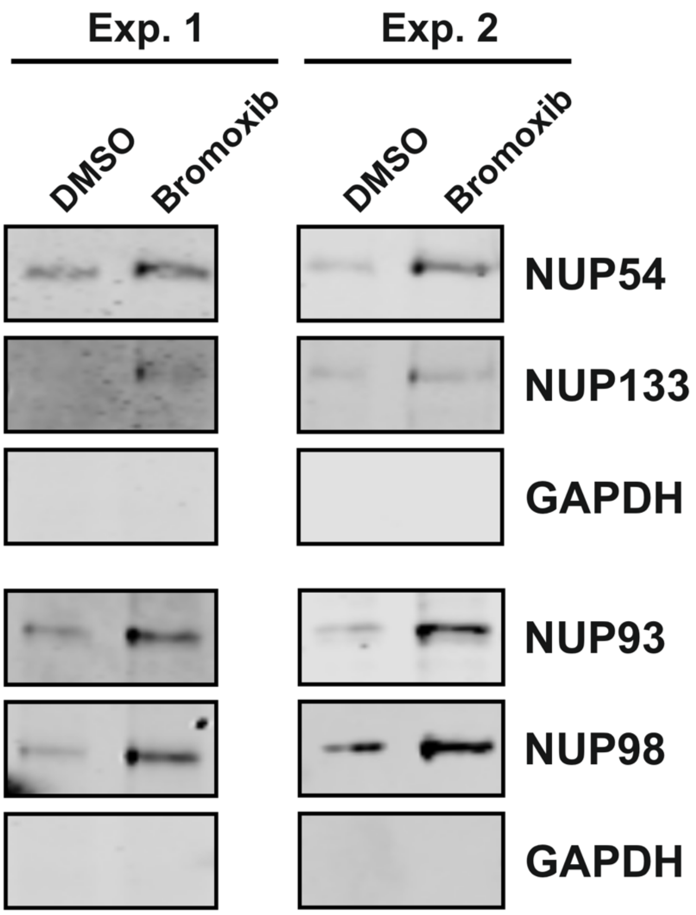

Suppl. Figure 2. Bromoxib mediates the aggregation of nucleoporins (NUPs).

Ramos cells were treated with DMSO (0.1% v/v) or Bromoxib (40 μM) for 30 min. Subsequently, cells were lysed using the standard lysis protocol and pellets were boiled in Laemmli buffer to reveal potential protein aggregates. Pellet samples were immunoblotted in the same way as samples in Figure 2. Immunoblots of two separate experiments ("Exp. 1" and "Exp. 2") are shown.
